# Longitudinal axial flow rice thresher feeding rate monitoring based on force sensing resistors

**DOI:** 10.1038/s41598-021-04675-w

**Published:** 2022-01-25

**Authors:** Mohamed Anwer Abdeen, Gan Xie, Abouelnadar Elsayed Salem, Jianwei Fu, Guozhong Zhang

**Affiliations:** 1 College of Engineering, Huazhong Agricultural University, Wuhan, 430070 China; 2 Agricultural Engineering Department, Zagazig University, Zagazig, 44519 Egypt; 3 Desert Research Center, Mataria, 11753 Egypt

**Keywords:** Sensors and probes, Engineering, Mechanical engineering

## Abstract

The threshing unit is the main working unit of the combine harvester and plays an essential role in rice threshing efficiency, seed loss, and damage. Every thresher has its limitation for feeding, and when the feeding quantity exceeds the maximum rated amount, the thresher gets blocked, resulting in higher losses, low threshing efficiency, more power consumption, and combine overloading shutting down. This study constructed a longitudinal axial flow rice threshing platform, and a stress monitoring system for the threshing drum top cover was designed using force sensing resistors. The sensors were installed on the thresher top cover inner surface to detect the impact and extrusion forces caused by the threshing process and detect the feeding rate when it exceeds the suitable feeding. Three feeding rates (0.8, 1.1, and 1.4 kg/s) and three thresher speeds (1100, 1300, and 1500 rpm) were tested. The time of the testing process was calculated using high-speed photography. The obtained results revealed that the force signals collected by thin-film sensors significantly correlated with thresher rotating speed and feeding rate. The thresher top cover’s average stress, average strain, and average total deformation were simulated using ANSYS finite element analysis. This study provides a new method for threshing drum real-time feeding quantity monitoring and early warning of thresher blockage.

## Introduction

Rice is the second important cereal in the world today after wheat, providing together 95 percent of the total staple food of the world’s population. The rice planting area in China is approximately 30 million hectares^1^ and is mainly harvested with combine harvesters^2,3^.

The grain harvester is vital agricultural machinery that improves harvesting efficiency and reduces labor costs^4–6^. The typical grain harvester combines the harvesting processes such as gathering, cutting, threshing, separation, cleaning, especially threshing is its most crucial function^7^. Many small, medium, and large hand-held and pedal-operated threshers have been used for a long time. However, they have not been implemented significantly because of their low performance compared to traditional methods^8^. Combine harvesters are a major force in rice harvesting in China, and the threshing and the cleaning device play an important role in the entire harvesting process^9,10^.

The threshing unit is the core device of the combine harvester, which determines the operating performance of the whole machine. Mechanical threshers are classified into axial-flow and cross-flow threshers. In axial-flow threshers, the crop moves along the axe of the cylinder, and it is subjected to multiple impacts from the cylinder. In cross-flow threshers, the crop is threshed while it moves between the cylinder and the concave transversally. In spike-tooth thresher, an array of spikes arranged along with the cylinder impacts the crop on the concave, wherein the rasp bar, a flat-surface cylinder runs above the concave, and the centrifugal force of the cylinder causes the crop to be threshed^11^. During threshing, rice stalks are fed along the axis of the cylinder, and the sloughed material is discharged from the opposite end. Because of the concave screen and the grass guide on the cover of the cylinder, the crop entering the thresher circulates around the cylinder during moving axially. The threshing of grain is caused by the threshing teeth impact on crop ears^12^. With the movement of the roller, the removed grain and straw are separated then discharged to the cleaning device for further separation.

The speed of the threshing drum influences the capacity and performance of a thresher^13^. An optimum speed is desirable for improved performance of the thresher because excessive speed can cause the grain to crack, while a low speed can give an unthreshed head. Due to unstable walking speed, crop lodging, maturity, water content, and inconsistent field growth state, the real-time feed amount of the harvester fluctuates during harvest. When the real-time feeding quantity exceeds the rated feeding quantity, the threshing drum is prone to overload, blockage, and shutting down, which may cause severe damage to the harvesting parts and seriously affect production efficiency^14^. Therefore, monitoring the real-time feeding quantity of rice combine harvester using force sensing resistors and early warning of the blockage of threshing drum is crucial to prevent the blockage of threshing drum.

Force sensing resistors are polymer thick film devices that exhibit a resistance decrease with increased applied force to its surface. They are simple to use and low in cost and detect physical pressure, squeezing, and weight. Piezoresistive sensors convert the external pressure into electrical signals utilizing resistance. Because of its simple technology, piezoresistive sensors have been applied in robots^15^, coal mine^16^, blasting^17,18^, medical treatment^19^, and other fields.

Xionget al.^20^ adopted piezoresistive thin-film sensor combined with the corresponding control system to realize the grip control of the manipulator. Wang et al.^21^ used piezoresistive thin-film sensors to measure the pressure of crushed corn stalks on helical conveying blades. Peng^22^ designed the plantar pressure distribution detector with a piezoresistive thin-film sensor, which can effectively detect the plantar pressure distribution.

Too low feeding rate reduces the separation rate, and too high feeding rate not only reduces the separation rate but also increases the un-threshed rate. By selecting a reasonable feed rate, it is possible to increase the separation rate while reducing the un-threshed rate^12^.

Excessive feeding rate and low thresher rotating speed can overload the thresher and lead to more losses, less threshing efficiency, more required power, and combine shutting down, which will increase threshing time and affect productivity and grain quality. Also, the change of rotational speed and feeding quantity of the thresher will change the force acting on its top cover. So that, a testing platform for longitudinal axial flow rice thresher was designed, and pressure film sensors were used as the main testing tool to detect the forces acting on the thresher top cover and monitor the thresher’s real-time feeding rate and early predict the blockage during the threshing process.

## Materials and methods

### Testing platform

**Figure 1:**
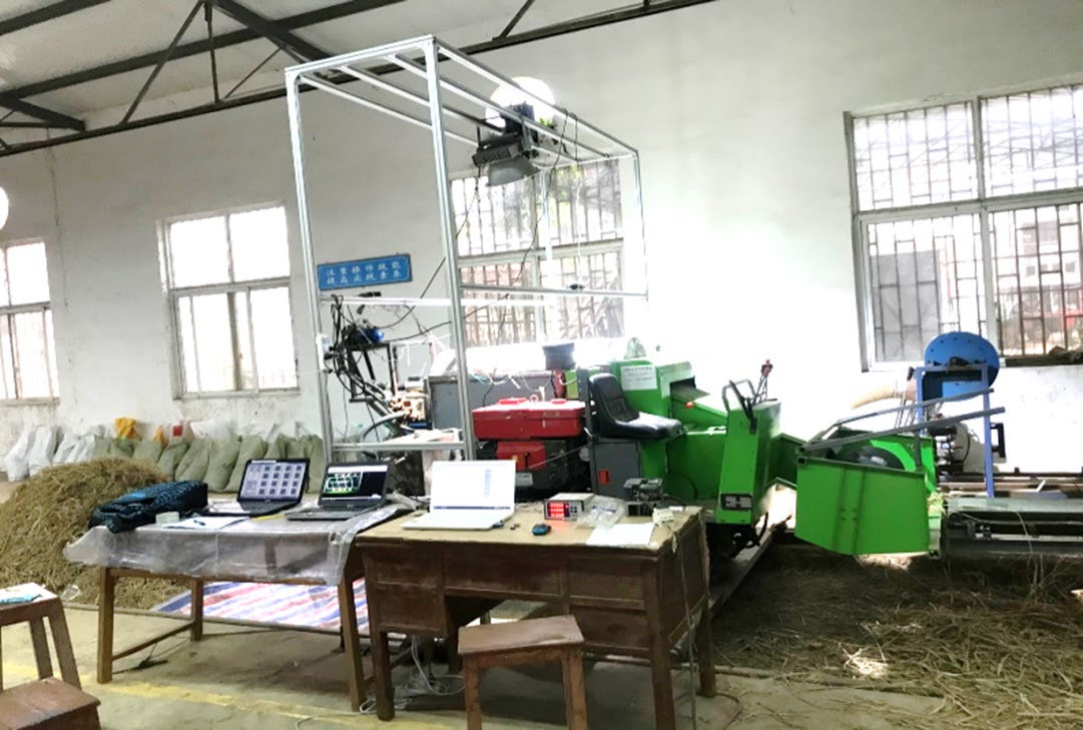
Testing platform.

The longitudinal axial flow testing platform with dimensions of (3700, 1460, and 1540 mm) for length, width, and height was constructed in the Engineering College factory, Huazhong agricultural university, Wuhan, China. The platform consisted of a thresher with spike teeth, concave, receiving boxes, cover with a transparent observation window, conveying belt, diesel engine, frequency convertor, feeding device, electric motor, pressure sensing system, torque sensor, and high-speed camera, as shown in (Fig. 1).

### Conveying mechanism

This mechanism composed of a revolving belt with dimensions of (6 × 0.5 m). The feeding device was driven by an electric motor using a pulley and belt. This mechanism was used to convey the rice to the feeding auger**.**

### Feeding auger

The feeding auger consisted of a rotating auger and a rotating chain with transverse steel bars. It was used to feed the rice from the conveyer to the threshing drum.

### Threshing device

**Figure 2:**
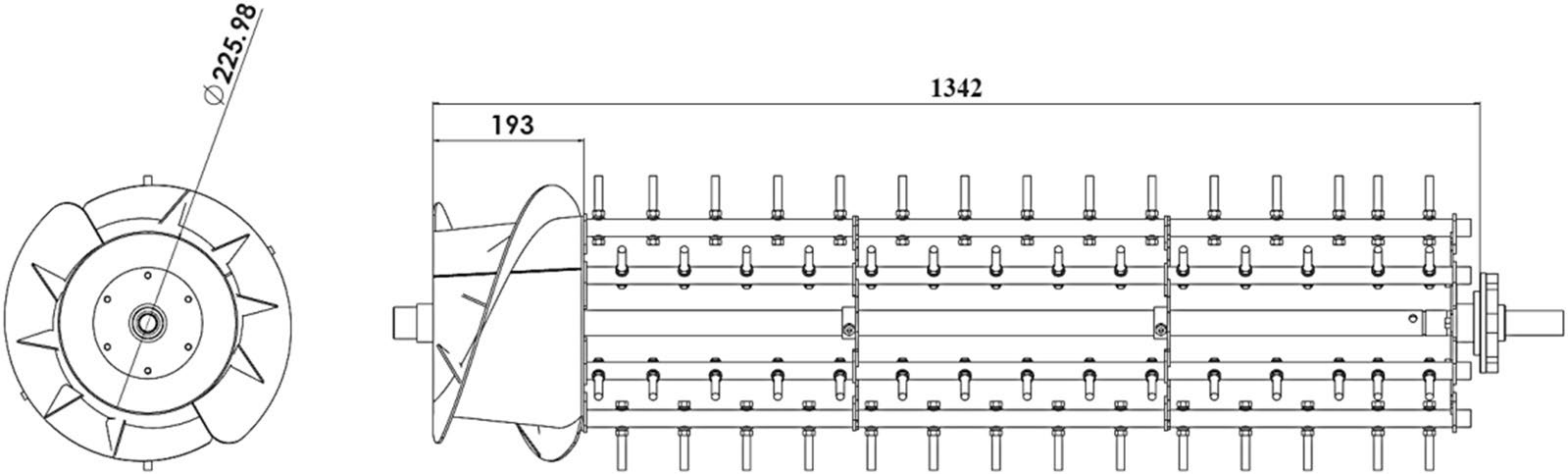
Longitudinal axial flow thresher with spike teeth (dimensions in mm).

The threshing device consisted of a cylindrical axial flow thresher with rod teeth, thresher cover with helical blades, and a perforated concave. The thresher was driven by a diesel engine using belts and pulleys. The thresher composes of 6 bars with spike teeth, as shown in (Fig. 2).

### Force testing system

**Figure 3:**
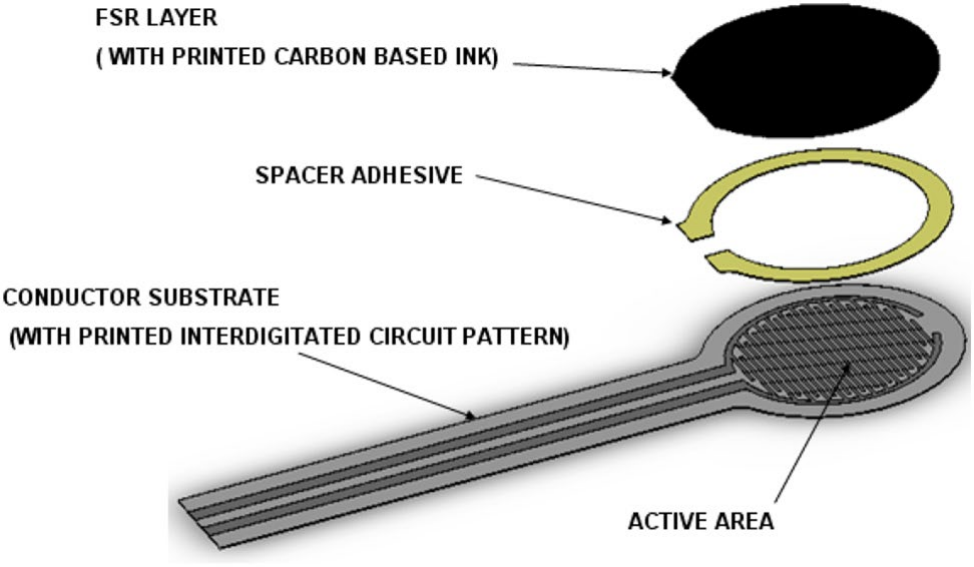
Force-sensing resistor.

Force Sensing Resistors (FSR) (Fig. 3) are devices that allow measuring static and dynamic forces applied to a contact surface. Their range of responses depends on the variation of their electric resistance^23^.

**Figure 4:**
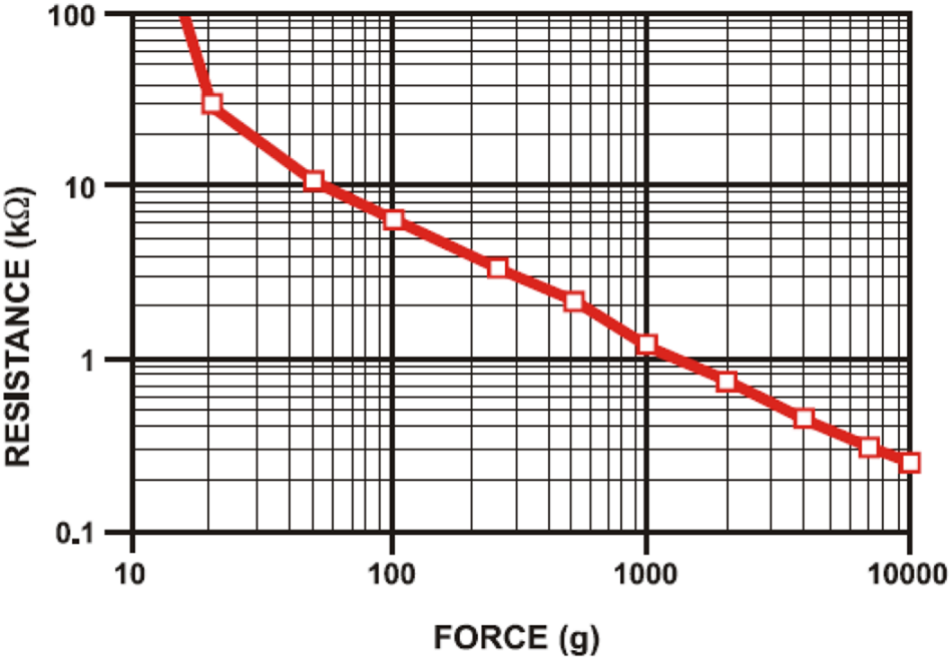
Resistance vs. force.

In the testing system, force-sensing resistors were used as the primary testing tool. These sensors are a polymer thin-film device that exhibits a decrease in the resistance with an increase in the force applied to its active surface, as shown in (Fig. 4).

**Figure 5:**
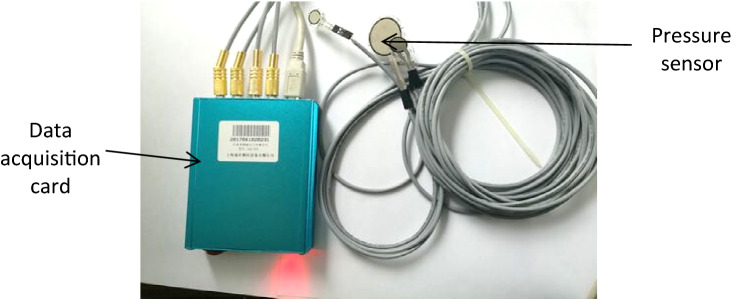
Pressure sensing system.

The pressure sensing system (Fig. 5) is a multichannel acquisition system consisting of two parts; hardware and software. The hardware system includes piezoresistive thin-film sensors, connecting cables, a data acquisition card, and a computer with Flexiforce software^24^. The software system consists of a data connection module, parameter setting module, data reading and display module, and data saving module.

**Figure 6:**
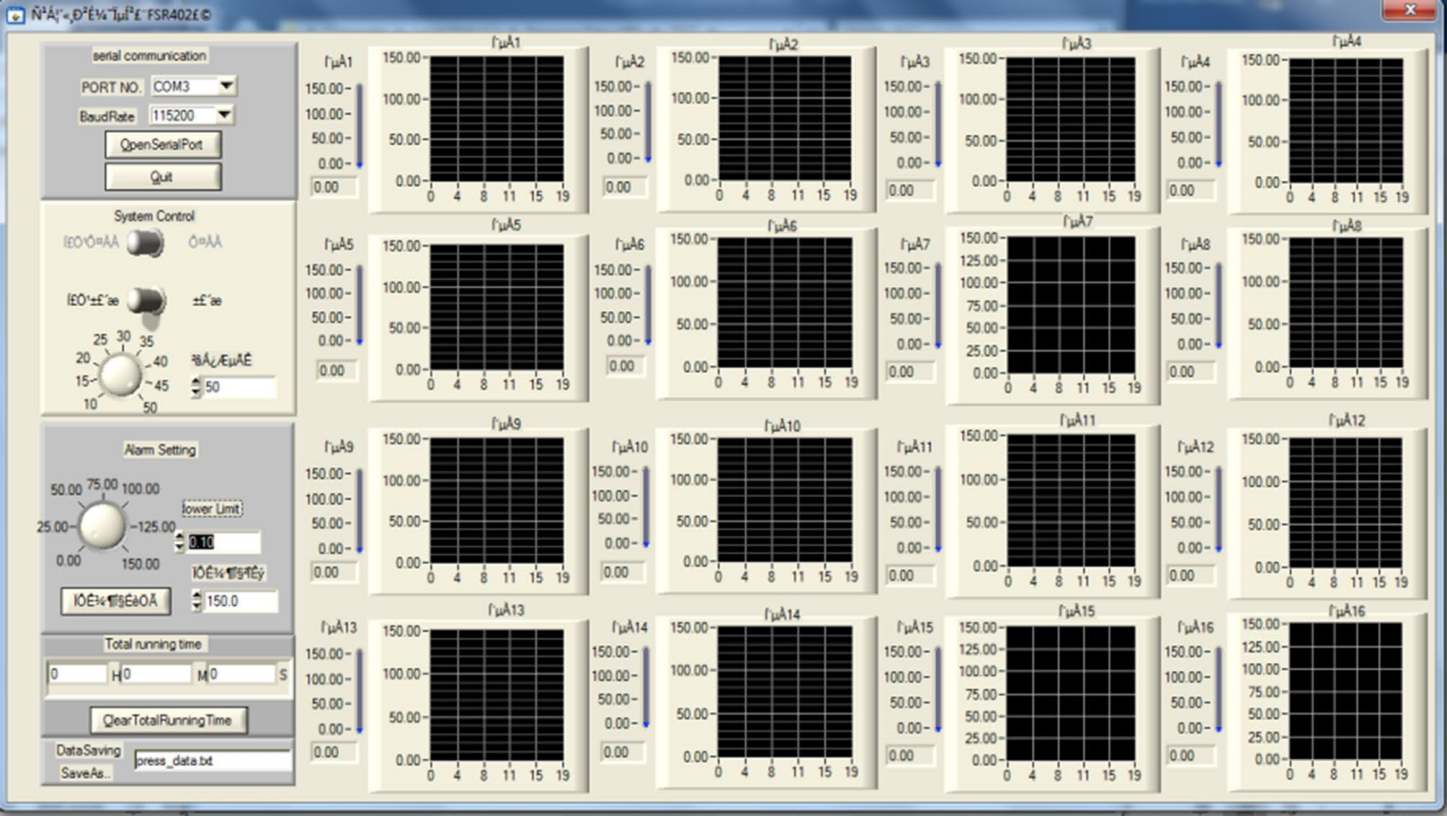
Software front panel.

The film sensors convert the pressure force into an analog resistance signal. The data acquisition card amplifies the resistance signal, converts it into digital signals, and transmits them to the Flexiforce software on the computer through the USB signal wire. The front panel of Flexiforce software is shown in (Fig. 6).

The test system can be started and stopped manually and also manually control data displaying and saving. The measured data collected by this system is saved in Newton as a data table in word pad or Excel format.

### Force sensor descriptions and dimensions

Model: 402, Active Area: 0.5″ [12.7 mm] diameter, Nominal thickness: 0.018″ [0.46 mm], Semi-conductive Layer: 0.005″ [0.13 mm] Ultem, Spacer Adhesive: 0.006″ [0.15 mm] Acrylic, Conductive Layer: 0.005″ [0.13 mm] Ultem, Rear Adhesive: 0.002″ [0.05 mm] Acrylic.

Sensors measured force ranges from 0 up to 150 N, and the force resolution of FSR devices is better than ± 0.5% of full use force.

### Sensor’s installation

**Figure 7:**
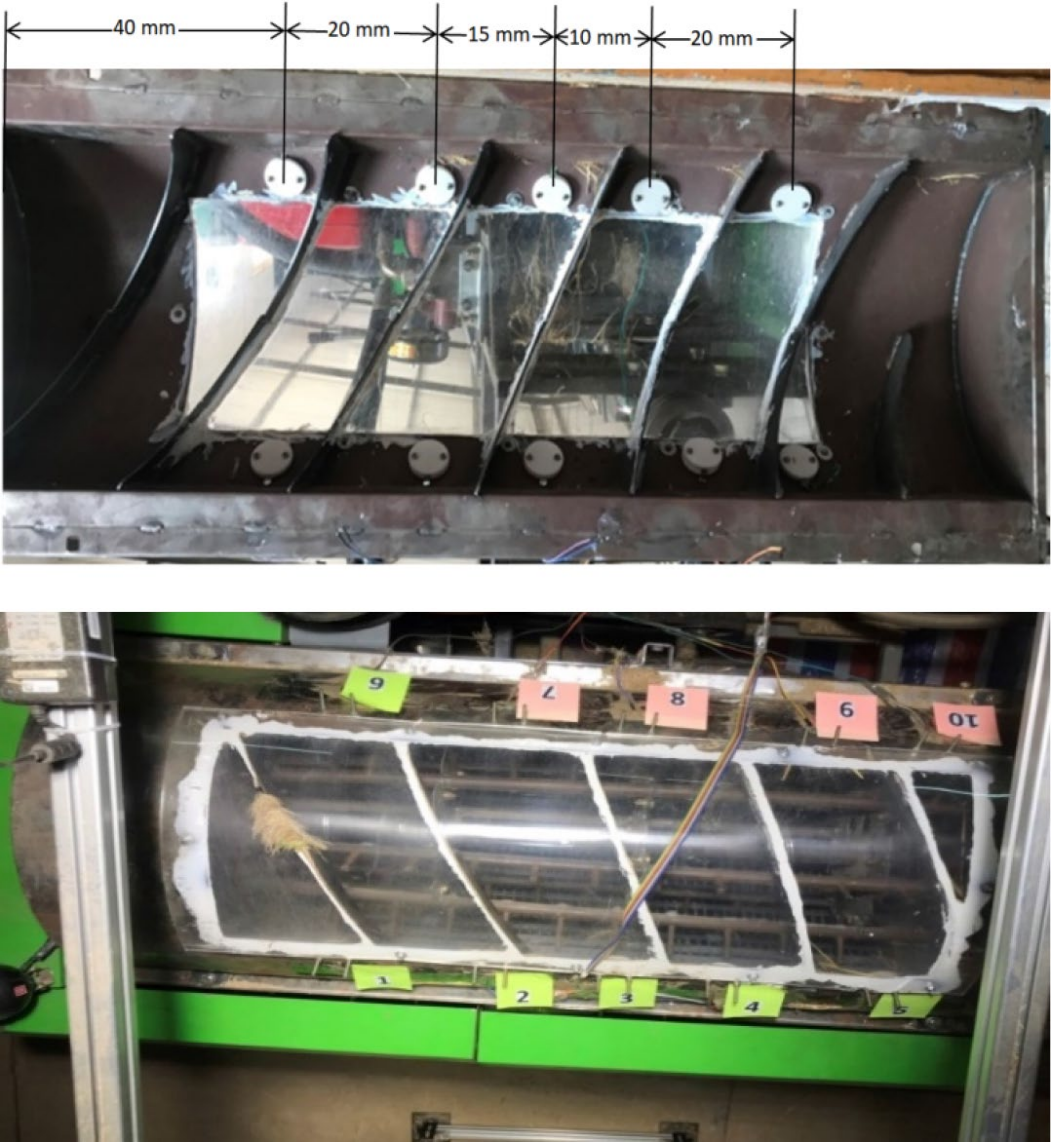
Sensors installation in thresher cover.

The sensors were installed on both sides of the thresher cover inner surface along its axis, as shown in (Fig. 7), so they can sense only the impact force from rice extrusion during the threshing process and neglect any disruption caused by machine vibration.

**Figure 8:**
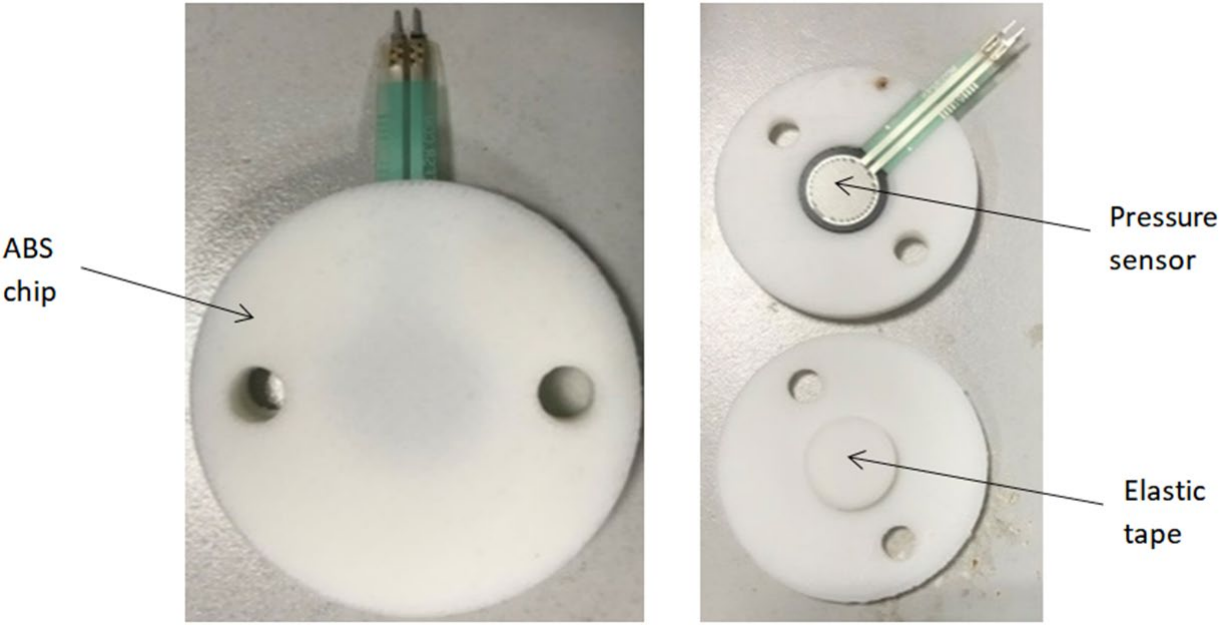
Installation of the sensors between ABS chips.

To guarantee the stability of the measured data, assure that the sensors would successfully feel the force under the elastic deformation, avoid signal disruption caused by poor contact, and to fix the sensors on the thresher top cover, ABS round chips (Fig. 8) were designed using Solidworks premium 2016 SP. 5^25^ and printed using a 3D printer, and every sensor placed between every two chips, these chips can also protect the sensors from damage.

An elastic tape was installed below and above every sensor to guarantee the elastic deformation, which gives resistance change.

### High-speed photography

**Figure 9:**
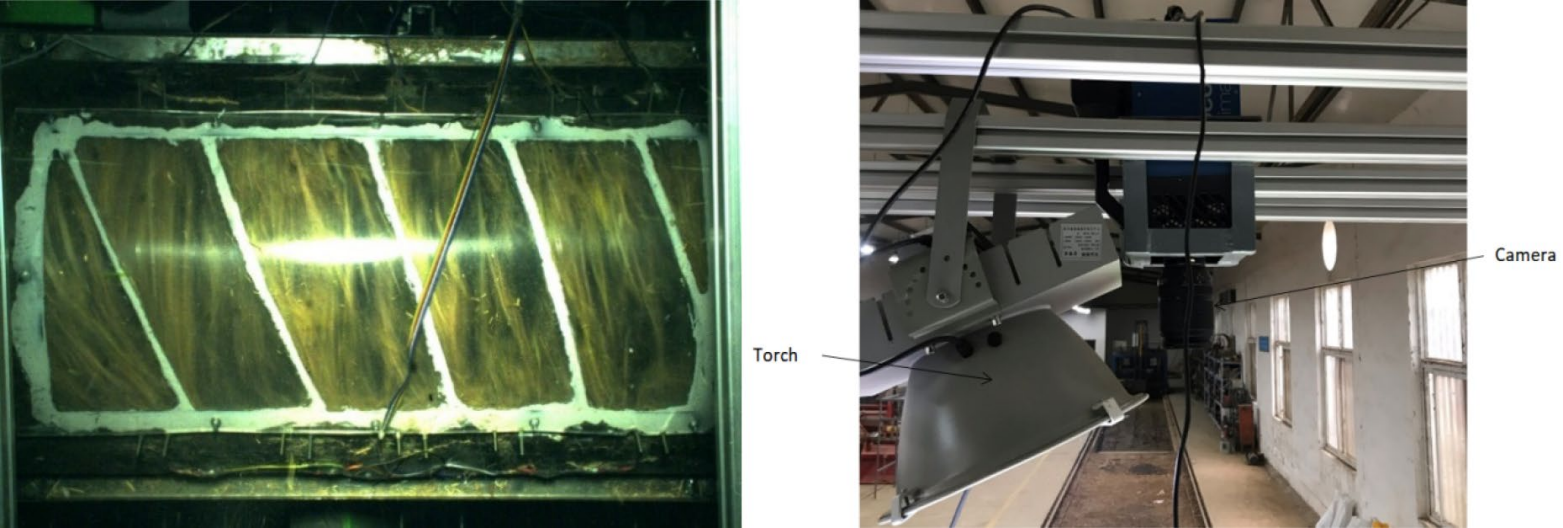
High-speed photography.

The feeding time of rice in the thresher was determined employing a high-speed camera (Fig. 9). The high-speed camera type was PCO dimax H.D. manufactured by PCO company, Germany, and the camera lens was A.F. micro-Nikon 60 mm f/2.8 manufactured by Nikon. The camera was fixed to a frame that could be moved along the axis of the platform. The photographic distance was set to 1500 mm, and the sampling frequency was set to 200 photos per second.

A window of 800 mm × 300 mm was opened on the thresher top cover and covered with 5 mm thick transparent Plexiglas for easy observation of rice movement.

### Testing procedure

The use of the plant in the current study complies with international, national, and institutional guidelines.

**Figure 10:**
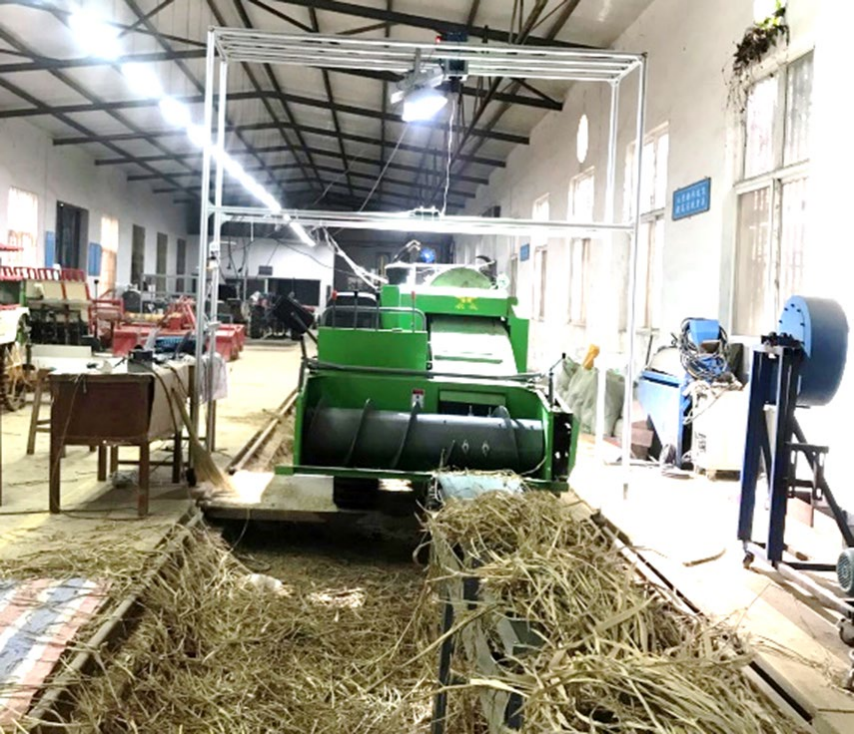
Testing procedure.

The threshing platform (Fig. 10) was constructed and experimented at Huazhong Agricultural University Engineering College’s factory. The conveyor length was 6 m, no rice was added to the first meter of the conveyor, and the rice crop regularly spread in the last 5 m to ensure it would be fed at a constant speed.

Preliminary experiments have been carried out to determine the maximum feeding rate. Three feeding rates of 0.8, 1.1, and 1.4 kg/s were tested as the maximum feeding rate of the thresher was 1.4 kg/s, and the drum speeds were 1100, 1300, and 1500 rpm.

## Results and discussion

After the test was carried out according to the aforementioned methods, the test data were collected and analyzed. High-quality graphs were drawn using OriginPro 2019b (64-bit) 9.6.5.169^26^, and ANOVA was carried out using Minitab 2017, 18.1^27^.

The forces measured by sensors 5 and 10 were too small, so they have been neglected.

The experiment design and results are shown in Table 1.

**Table 1:** Experimental results.

Feeding rate, kg/s	Thresher speed, rpm	Measured force, N							
		Sensor 1	Sensor 2	Sensor 3	Sensor 4	Sensor 6	Sensor 7	Sensor 8	Sensor 9
0.80	1100	0.01	0.09	0.02	2.15	0.01	0.42	2.01	0.35
1.10	1100	0.02	0.19	0.09	2.22	0.02	0.83	2.31	0.54
1.40	1100	0.08	0.23	0.64	2.99	0.08	1.06	3.00	1.05
0.80	1300	0.06	0.90	0.69	2.70	0.06	0.85	2.12	0.64
1.10	1300	0.15	0.90	0.89	3.18	0.15	1.03	3.02	1.45
1.40	1300	0.23	1.07	1.14	3.57	0.24	1.76	3.64	1.65
0.80	1500	0.70	1.15	2.19	3.37	0.73	1.81	3.01	1.12
1.10	1500	1.02	1.17	2.32	3.45	1.06	2.56	3.30	1.56
1.40	1500	1.11	1.28	2.74	3.78	1.10	2.88	3.82	1.99

### Effect of feeding rate on pressure sensors data under different thresher speeds

**Figure 11:**
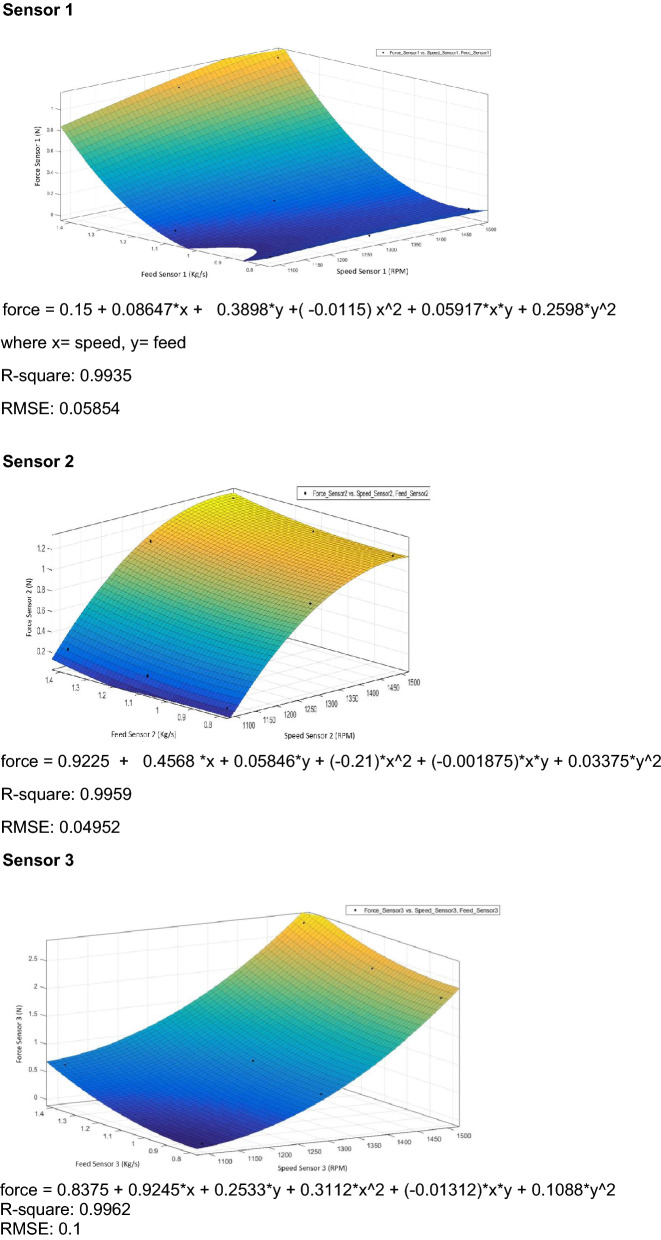

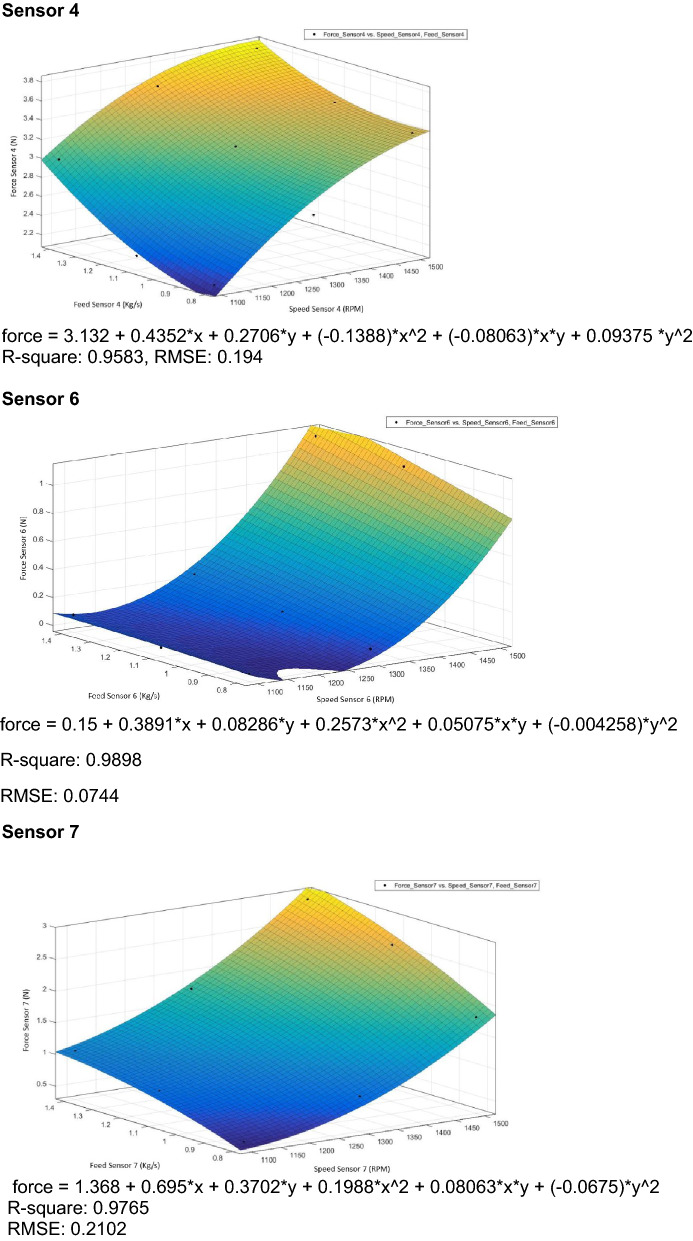

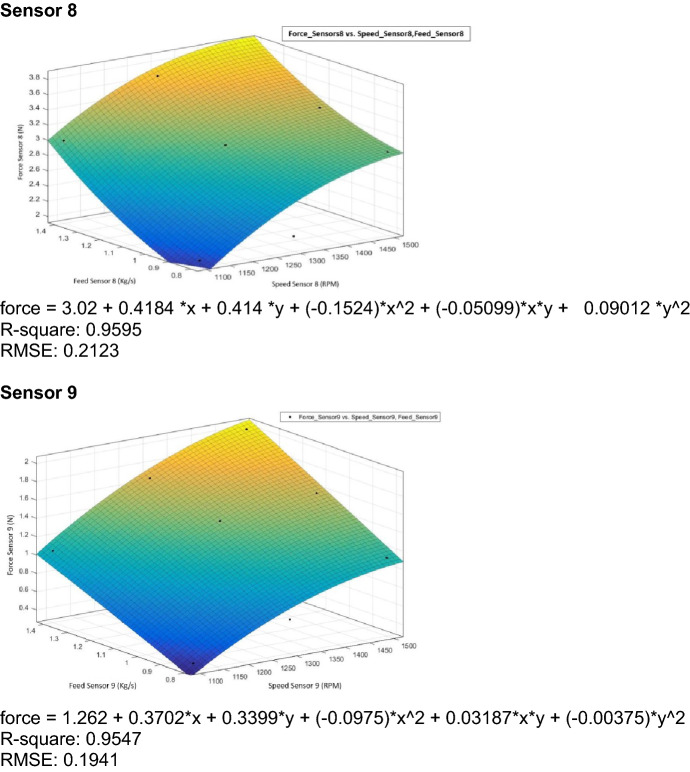
Force measured by pressure film sensors.

The obtained results (Fig. 11) showed a positive relationship between the feeding rate and the measured forces for all the sensors at every thresher speed. This may be attributed to increasing crop density and crop layer thickness in the threshing gap, which results in high impact force and pressure on the thresher top cover and sensors. This result was the same as the obtained result by Shenghua et al.^28^, who concluded that increasing the feeding rate for a horizontal axial threshing drum increased the force acting on the thresher top cover.

### Effect of thresher speed on pressure sensors data under different feeding rates

**Figure 12:**
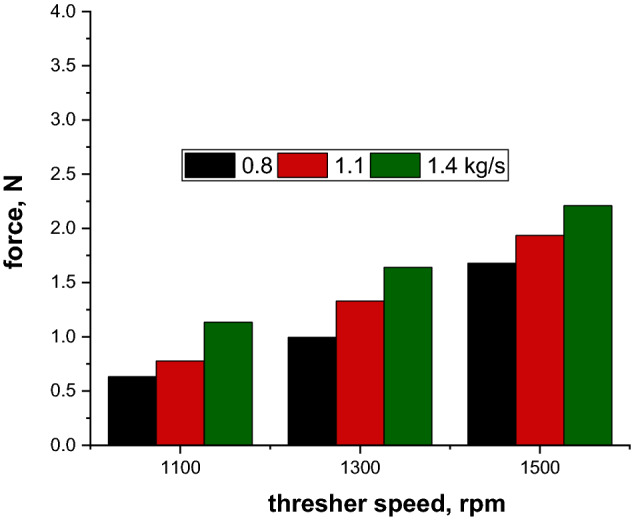
Effect of thresher speeds on pressure sensors data under different feeding rate.

It was observed that increasing thresher speed tended to increase the force measured by the pressure film sensors for all the feeding rates (Fig. 12). This might be attributed to the increase in the collision force of rice stalks to the pressure sensors and thresher top cover. This result was in agreement with the result obtained by Shenghua et al.^28^, who concluded that increasing the threshing speed of a horizontal axial threshing drum increased the force acting on the thresher top cover.

### Analysis of variance (ANOVA)

ANOVA is a statistical method used for determining the individual interactions of every control factor in the testing design. It was used to analyze the effect of thresher rotating speed and feeding rate on the forces measured by the pressure sensors. The analysis was carried out at a 5% significance level and a 95% confidence level. ANOVA showed that feeding rate and thresher speed significantly affected the force measured by pressure sensors.

Results are shown in (Tables 2, 3, 4, 5, 6, 7, 8, 9).

**Table 2:** Analysis of variance sensor 1.

Source	DF	Seq SS	Contribution (%)	Adj SS	Adj MS	F-value	P-value
Feed rate	2	0.07215	4.54	0.07215	0.036076	3.69	0.124
Thresher speed, rpm	2	1.47693	92.99	1.47693	0.738465	75.50	0.001
Error	4	0.03912	2.46	0.03912	0.009781		
Total	8	1.58820	100.00

**Table 3:** Analysis of variance sensor 2.

Source	DF	Seq SS	Contribution (%)	Adj SS	Adj MS	F-value	P-value
Feed rate	2	0.03215	1.81	0.03215	0.016077	13.54	0.017
Thresher speed, rpm	2	1.74218	97.93	1.74218	0.871089	733.61	0.000
Error	4	0.00475	0.27	0.00475	0.001187		
Total	8	1.77908	100.00

**Table 4:** Analysis of variance sensor 3.

Source	DF	Seq SS	Contribution (%)	Adj SS	Adj MS	F-value	P-value
Feed rate	2	0.47702	6.06	0.47702	0.23851	42.95	0.002
Thresher speed, rpm	2	7.37466	93.66	7.37466	3.68733	664.06	0.000
Error	4	0.02221	0.28	0.02221	0.00555		
Total	8	7.87389	100.00

**Table 5:** Analysis of variance sensor 4.

Source	DF	Seq SS	Contribution (%)	Adj SS	Adj MS	F-value	P-value
Feed rate	2	0.7957	29.27	0.7957	0.39787	13.30	0.017
Thresher speed, rpm	2	1.8032	66.33	1.8032	0.90162	30.13	0.004
Error	4	0.1197	4.40	0.1197	0.02993		
Total	8	2.7187	100.00

**Table 6:** Analysis of variance sensor 6.

Source	DF	Seq SS	Contribution (%)	Adj SS	Adj MS	F-value	P-value
Feed rate	2	0.06755	4.13	0.06755	0.033776	3.76	0.120
Thresher speed, rpm	2	1.53067	93.67	1.53067	0.765333	85.25	0.001
Error	4	0.03591	2.20	0.03591	0.008977		
Total	8	1.63413	100.00

**Table 7:** Analysis of variance sensor 7.

Source	DF	Seq SS	Contribution (%)	Adj SS	Adj MS	F-value	P-value
Feed rate	2	1.1515	20.44	1.1515	0.57573	17.25	0.011
Thresher speed, rpm	2	4.3494	77.19	4.3494	2.17469	65.15	0.001
Error	4	0.1335	2.37	0.1335	0.03338		
Total	8	5.6343	100.00

**Table 8:** Analysis of variance sensor 8.

Source	DF	Seq SS	Contribution (%)	Adj SS	Adj MS	F-value	P-value
Feed rate	2	1.8329	55.06	1.8329	0.91647	20.53	0.008
Thresher speed, rpm	2	1.3176	39.58	1.3176	0.65882	14.76	0.014
Error	4	0.1785	5.36	0.1785	0.04464		
Total	8	3.3291	100.00

**Table 9:** Analysis of variance sensor 9.

Source	DF	Seq SS	Contribution (%)	Adj SS	Adj MS	F-value	P-value
Feed rate	2	1.11546	44.77	1.11546	0.55773	22.90	0.006
Thresher speed, rpm	2	1.27862	51.32	1.27862	0.63931	26.25	0.005
Error	4	0.09742	3.91	0.09742	0.02435		
Total	8	2.49149	100.00				

### Regression analysis

Regression analyses are used for analyzing many variables when there is a relationship between a dependent variable and one or more independent variables^29^. The dependent variable is the force measured by the pressure sensors, and the independent variables are feed rate and thresher speed.

The linear regression equations are given below, where:

- Y_1100_: Force measured during thresher speed of 1100 rpm.
- Y_1300_: Force measured during thresher speed of 1300 rpm.
- Y_1500_: Force measured during thresher speed of 1500 rpm.
- X: Feeding rate.

**Table:** 

Regression equations sensor 1 Y_1100_ = − 0.361 + 0.361 X Y_1300_ = − 0.250 + 0.361 X Y_1500_ = 0.548 + 0.361 X	Regression equations sensor 2 Y_1100_ = − 0.0897 + 0.2351 X Y_1300_ = 0.6968 + 0.2351 X Y_1500_ = 0.9417 + 0.2351 X
Regression equations sensor 3 Y_1100_ = − 0.741 + 0.904 X Y_1300_ = − 0.086 + 0.904 X Y_1500_ = 1.421 + 0.904 X	Regression equations sensor 4 Y_1100_ = 1.153 + 1.182 X Y_1300_ = 1.850 + 1.182 X Y_1500_ = 2.235 + 1.182 X
Regression equations sensor 6 Y_1100_ = − 0.341 + 0.344 X Y_1300_ = − 0.226 + 0.344 X Y_1500_ = 0.585 + 0.344 X	Regression equations sensor 7 Y_1100_ = − 0.837 + 1.460 X Y_1300_ = − 0.392 + 1.460 X Y_1500_ = 0.809 + 1.460 X
Regression equations sensor 8 Y_1100_ = 0.418 + 1.840 X Y_1300_ = 0.900 + 1.840 X Y_1500_ = 1.355 + 1.840 X	Regression equations sensor 9 Y_1100_ = − 0.929 + 1.434 X Y_1300_ = − 0.331 + 1.434 X Y_1500_ = − 0.021 + 1.434 X

### Simulation of the forces acting on thresher top cover using finite element analysis

Finite element analysis (FEA) is a numerical method for solving specific problems in engineering and science. These methods are needed because analytical methods cannot cope with the complicated problems that are met within engineering. One of FEA’s first applications was to find the stresses and strains in engineering components under load.

The finite element analysis has been used to analyze cylindrical objects. For example, Rawat et al.^30^ used the finite element method to perform a modal analysis on a closed thin-walled cylindrical shell, and the effect of the ratio of the end face thickness to the shell thickness on its natural frequency was studied.

**Figure 13:**
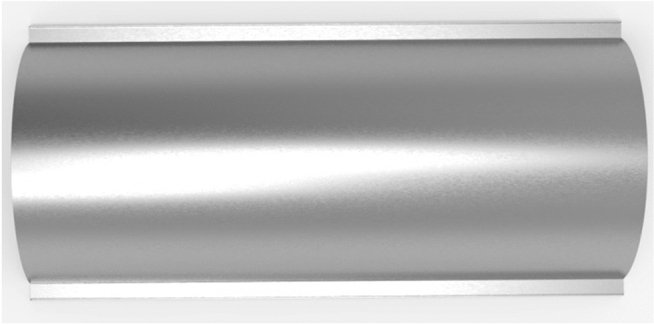
Thresher top cover 3D model.

In this study, the 3D model (Fig. 13) has been created using Solidworks premium 2016 SP. 5^25^, and saved in STEP format, and then the model imported to ANSYS v.12^31^ for the simulation.

**Figure 14:**
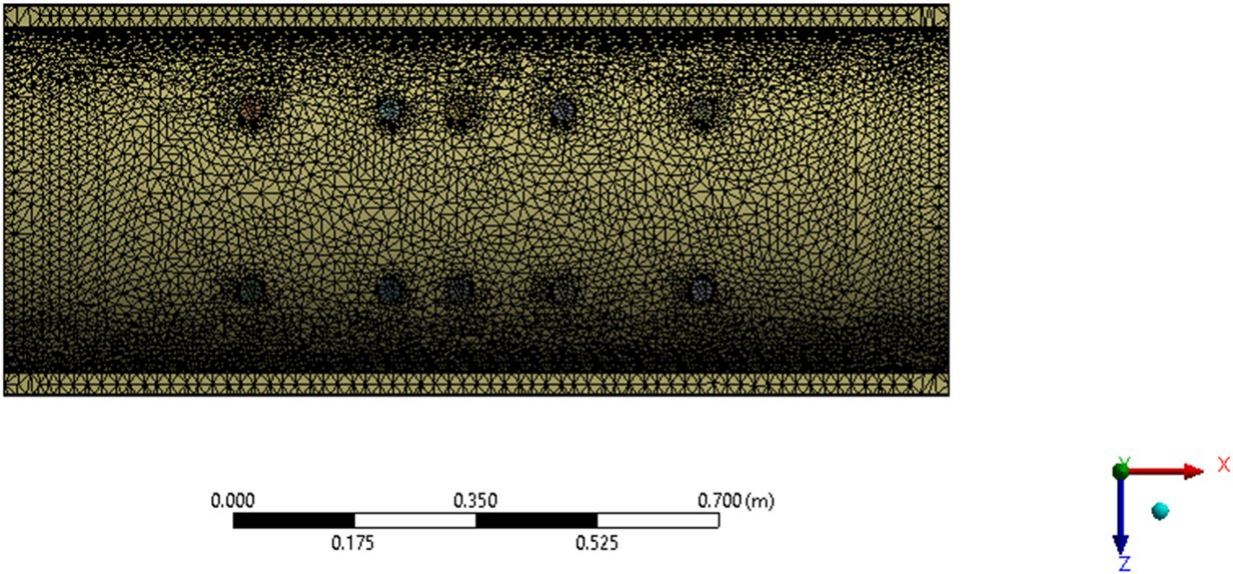
Meshing.

The meshing element size was set to 10 mm, as shown in Fig. 14.

**Figure 15:**
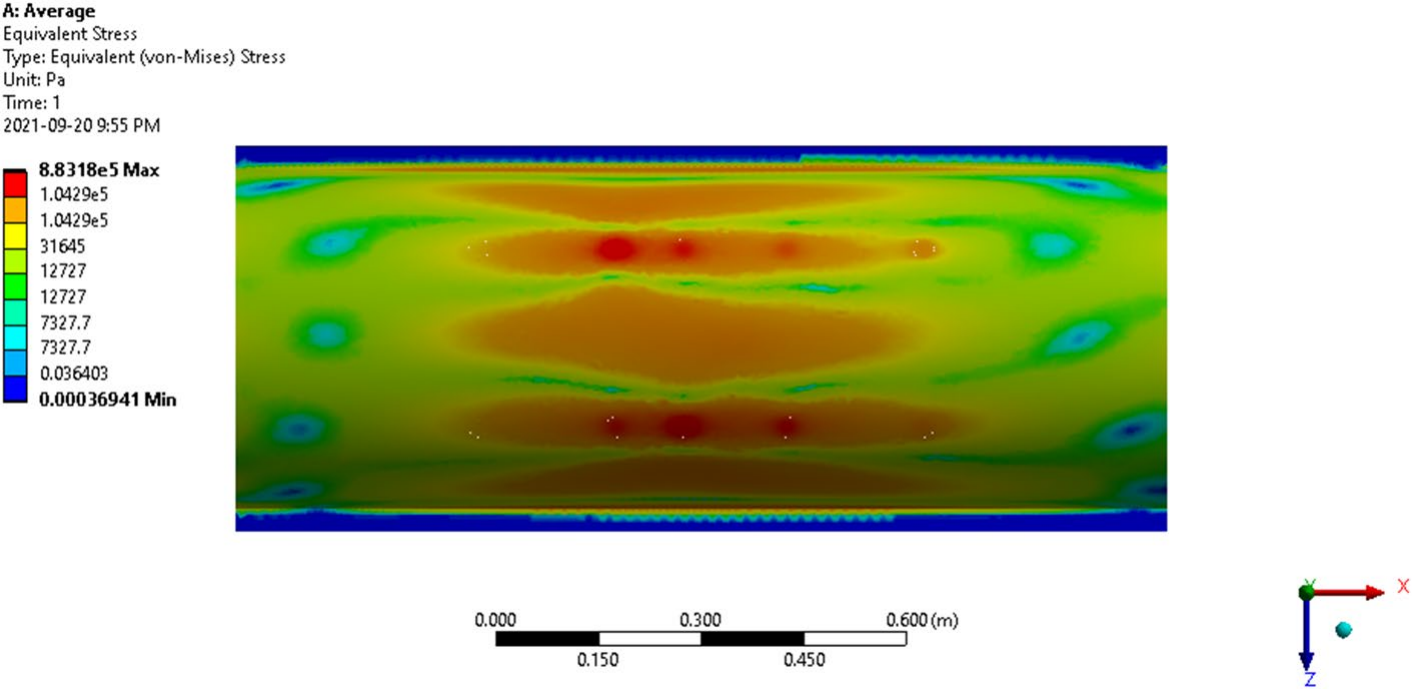
Average stress of the thresher top cover.

**Figure 16:**
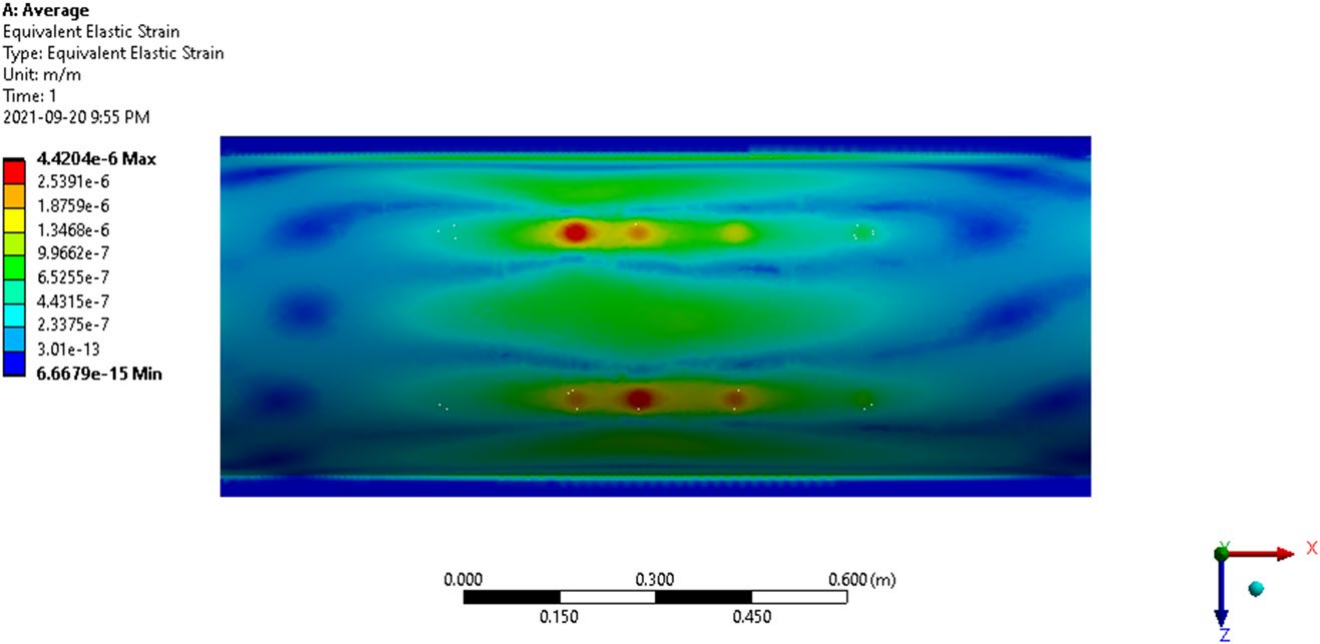
The average strain of the thresher top cover.

**Figure 17:**
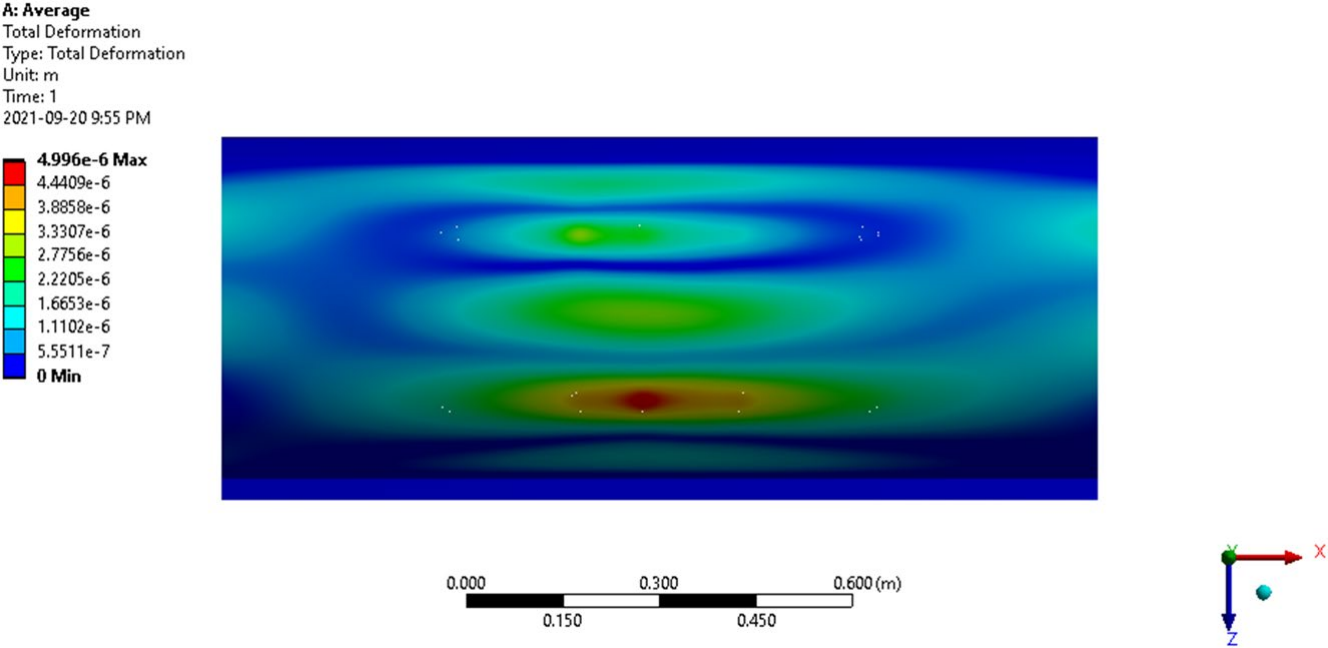
Average total deformation of the thresher top cover.

The thresher top cover’s average stress (Fig. 15), average strain (Fig. 16), and average total deformation (Fig. 17) were simulated using ANSYS using finite element analysis.

## Conclusion

In this paper, a stress monitoring system for the top cover of longitudinal axial flow rice threshing drum was designed depending on force-sensing resistors as the primary testing tool. The pressure sensors have been fixed on the inner surface of the thresher top cover along its axis using ABS chips. The pressure system was used to measure the impact and extrusion forces caused by rice crop during the threshing process and monitor the feeding rate of the thresher in seeking of predicting the combine overfeeding to avoid thresher blockage, which may cause severe damage to the harvesting parts and seeds and results in combine shutting down at the end.

The experiments were carried out under different thresher rotating speeds (1100, 1300, and 1500 rpm) and different feeding rates (0.8, 1.1, 1.4 kg/s).

A high-speed photographic bench was built, and the time of the testing process was determined using a high-speed camera which was fixed above the testing platform.

After the obtained data had been analyzed, it was revealed that there was a positive relationship between the force measured by the pressure sensors and thresher rotating speed and feeding rate as well. The thresher top cover’s average stress, average strain, and average total deformation were simulated using ANSYS finite element analysis.

This research study provides a new method for threshing drum real-time feeding quantity monitoring and early warning of its blockage using pressure film sensors, which will help in increasing threshing performance, decreasing threshing power and losses, and optimizing the rice combine performance.
